# Maximising the potential of administrative data to examine homelessness in Northern Ireland

**DOI:** 10.23889/ijpds.v8i2.2332

**Published:** 2023-09-14

**Authors:** Siobhán Murphy, Eileen Mitchell, Dermot O'Reilly

**Affiliations:** 1 Queen's University Belfast, Belfast, United Kingdom

## Objectives

Homelessness is a growing public health concern in Northern Ireland. Our study intends to measure the scale of homelessness in Northern Ireland and identify the potential of using linked administrative data to understand the complex needs of people who experience homelessness (PEH) and contribute to policy and service development.

## Methods

All formal applications for homelessness go to one central body the Northern Ireland Housing Executive (NIHE). However, it is possible that a proportion might remain unknown to local housing authorities due to personal circumstances, changes in legislation or eligibility criteria etc, which might mean that formal applications underestimate the true scale of homelessness. In this study of the interplay of homelessness health and exposure to social services, we can identify emergency accommodation centres by using ArcGIS, a geographic information system that allows users to analyse maps and spatial data to identify individuals who have been residing at hostels, addiction centres or women's refuges.

## Results

We identified 115 emergency accommodation centres. We plan to use this information to quantify the scale of homelessness in NI and compare the demographic profiles of those who may not be registered with local housing authorities. It is likely these individuals may represent a highly vulnerable population with complex needs and need tailored support packages.

## Conclusion

We will discuss some of the limitations of housing-administrative data and the methodologies we explored to help identify those people who may not be known to local authorities that are experiencing homelessness. Our findings can be used to inform policy on providing continuity of care and support for all people experiencing homelessness and reduce barriers to timely access of this support.

